# Improved Precision of COPD Exacerbation Detection in Night-Time Cough Monitoring

**DOI:** 10.3390/jpm15080349

**Published:** 2025-08-02

**Authors:** Albertus C. den Brinker, Susannah Thackray-Nocera, Michael G. Crooks, Alyn H. Morice

**Affiliations:** 1 Independent Researcher, NL-5708 DJ Helmond, The Netherlands; 2 Centre for Clinical Science, Hull York Medical School, University of Hull, Cottingham HU16 5JQ, UK; susannah.thackray-nocera@nhs.net (S.T.-N.); michael.crooks@nhs.net (M.G.C.); a.h.morice@hull.ac.uk (A.H.M.)

**Keywords:** COPD, exacerbation, alert, cough, rule-based classification, stratification

## Abstract

**Background/Objectives:** Targeting individuals with certain characteristics provides improved precision in many healthcare applications. An alert mechanism for COPD exacerbations has recently been validated. It has been argued that its efficacy improves considerably with stratification. This paper provides an in-depth analysis of the cough data of the stratified cohort to identify options for and the feasibility of improved precision in the alert mechanism for the intended patient group. **Methods:** The alert system was extended using a system complementary to the existing one to accommodate observed rapid changes in cough trends. The designed system was tested in a post hoc analysis of the data. The trend data were inspected to consider their meaningfulness for patients and caregivers. **Results:** While stratification was effective in reducing misses, the augmented alert system improved the sensitivity and number of early alerts for the acute exacerbation of COPD (AE-COPD). The combination of stratification and the augmented mechanism led to sensitivity of 86%, with a false alert rate in the order of 1.5 per year in the target group. The alert system is rule-based, operating on interpretable signals that may provide patients or their caregivers with better insights into the respiratory condition. **Conclusions:** The augmented alert system operating based on cough trends has the promise of increased precision in detecting AE-COPD in the target group. Since the design and testing of the augmented system were based on the same data, the system needs to be validated. Signals within the alert system are potentially useful for improved self-management in the target group.

## 1. Introduction

Telemonitoring for COPD is an active research area, with systematic reviews and meta-analyses appearing regularly [1,2,3,4,5,6,7,8]. An umbrella review [9] stated that telemonitoring interventions are associated with significant reductions in respiratory exacerbation and hospitalisation rates as one of its conclusions. The early detection of acute exacerbation (AE) enables prompt treatment, leading to improved exacerbation recovery, reductions in hospitalisation, and better health-related quality of life [10]. Since COPD is a heterogeneous lung condition [11,12], a personalised, patient-centric approach is necessary for effective management.

In COPD telehealth scenarios, various monitoring methods can be deployed. The traditional approach includes pulmonary function tests and daily questionnaires or diaries, while more recent methods include objective measurements like coughing [8], lung sounds [13], vital signs [14,15,16], and activity monitoring [17]. The monitoring method is important, as the success of telemonitoring depends on adherence, and adherence depends on the balance between the perceived burden and effectiveness. The monitoring should be hassle-free, informative, and highly specific to prevent additional burdens, alarm fatigue, and over-treatment. The use of stratification provides greater precision.

Cough monitoring is a well-known method for the monitoring of respiratory issues and has the advantage that truly hassle-free systems are available as night-time passive monitors. A cough monitor can provide relevant data to a caregiver but can also be leveraged for improved self-management. An alert or traffic light system may be helpful as pointers for the interpretation of cough count graphs, which is meaningful in view of the difficulty in recognising early symptoms of deterioration [14,18,19].

An alert system based on unobtrusive night-time cough monitoring has been validated recently [20]. As expected, using coughing as the only information source to trigger exacerbation alerts is insufficient: some exacerbations do not exhibit an increased number of coughs. It has been estimated that approximately 30% of exacerbations cannot be detected by cough monitoring. Fortunately, there are several strategies by which to select the appropriate cohort in order to prevent the use of monitors in patients where little or no effect is expected [21]. Stratification is considered crucial to establish trust in and acceptance of the monitoring system and to avoid an unnecessary healthcare burden.

In this paper, we consider the validated passive night-time cough in a post hoc analysis and propose an additional processing strategy to improve its performance. While stratification improves efficacy by reducing the number of misses (i.e., rejecting patients where the monitored data do not carry the relevant information), a complementary strategy is the improvement of sensitivity. The performance of a system using a combination of both strategies is discussed. Furthermore, it is noted that the alert mechanism is not a black box but an explainable rule-based system, enabling personalised medicine. Examples are provided to support the notion that signals within the system are interpretable for patients and caregivers. This implies the possibility of improved self-management for patients in the target group. With provisions for patient annotation in the system, this may pave the way for a refined, personalised alert system with settings or even cough patterns adapted to the patient.

## 2. Methods and Materials

This post hoc analysis is a sequel to the study described in [20], where the results regarding its primary aim were reported. Here, we cover aspects of the secondary aim. Since no new data were used, this section is largely a recapitulation, with the exception of the description of the augmented cough-based alert mechanism and the evaluation metrics relevant to the current aim.

### 2.1. Trial Set-Up

Where [20] covered the validation of a cough-based alert mechanism for AE-COPD, the current paper addresses the secondary aim of the trial: the improved precision of the validated alert mechanism. The study was reviewed and approved by the Internal Committee Biomedical Experiments of Philips Research and the North East—York Research Ethics Committee, United Kingdom Health Research Authority (REC Ref.: 21/YH/0203), with informed consent obtained from all participants involved in the study. The trial was a prospective longitudinal study of continual cough monitoring in COPD patients, which was executed in a double-blind fashion to prevent investigator bias. The patients were monitored for 12 weeks, and, if no exacerbation occurred in this period, they were asked to continue for a further 12 weeks. On a monthly basis, device operation was checked in the patient’s home, questionnaire data were collected, and incident report forms were created.

The cohort was set at 40 patients, where the inclusion criteria included a clinical diagnosis of COPD according to the NICE guidance [22], a smoking history of 10 packyears or more, and two or more moderate and/or severe exacerbations of COPD in the previous year. Exclusion criteria included significant comorbid medical or psychological conditions that were deemed by the principal investigator to affect the cough frequency or the subject’s ability to comply with trial procedures. Data from 32 patients were ultimately available for cough count analysis [23].

### 2.2. Data Processing

**Figure 1:**
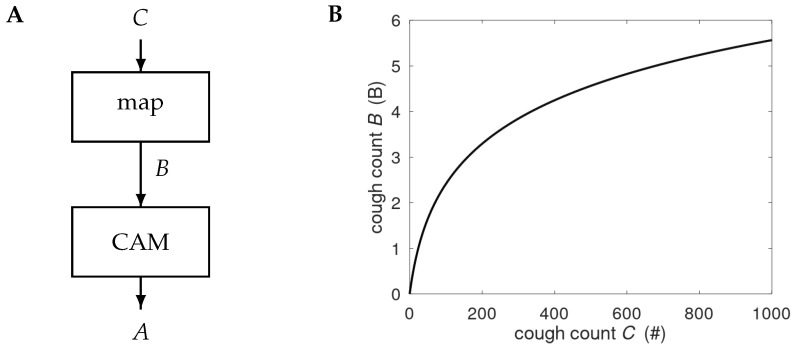
(**A**) Processing of the night-time cough count *C*. The counts are mapped onto the B-scale and processed by the cough-based alert mechanism (CAM) to produce alerts *A*. Details of the CAM are provided in Figure 2. (**B**) Graph showing the compressive nature of the map C→B, with # denoting the number of coughs observed in a night-time session.

Night-time cough counts were collected from COPD patients in their homes by a passive, stationary system placed in the sleeping quarters, typically on the bedside table closest to the patient. Coughs were extracted from the audio signal using a personalised classifier. This personalisation means that the system is adapted to the patient’s cough sounds, their cough etiquette [24], and the acoustic environment [23]. The monitoring sessions were run from 9 PM to 9 AM, and the total number of observed coughs in this period was denoted as *C*. The cough counts were mapped to the B-scale according to

(1) $$B=\alpha \log_{10}\left\{1+\beta C\right\},$$

with α=3.45 and β=0.04. Figure 1 depicts the high-level two-step processing C→B→A, where *A* denotes the raised alerts; it also visualises the logarithmic (compressive) mapping. The B-scale was designed as a pre-processing method, enabling patient-independent threshold settings in the alert mechanism [25].

### 2.3. Alert Mechanism

**Figure 2:**
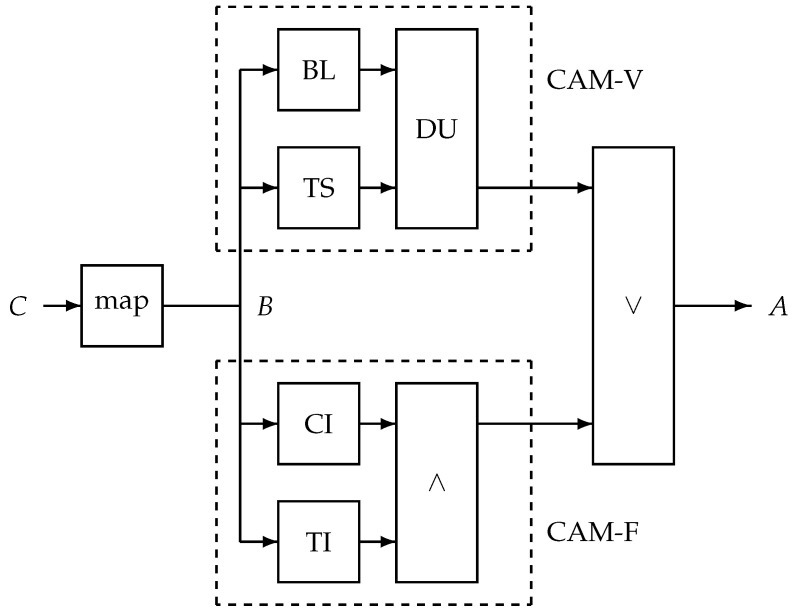
Proposed alert system with the time series *B* as input, a mapped version of the session cough count *C*. It processes *B* in two blocks: the validated unit CAM-V and the proposed fast-response unit CAM-F. The outputs of both systems are merged into alert *A* by a logical -OR operation (∨). The CAM-V units are as follows: BL—baseline creation; TS—temporal smoother; DU—decision unit. The CAM-F units are as follows: CI—unit to verify consistent increase; TI—unit to quantify total increase; ∧—logical -AND operation.

The proposed alert mechanism is shown in Figure 2 and consists of two parallel processing paths called CAM-V and CAM-F, where -V corresponds to the validated alert mechanism and -F to the proposed additional unit. The suffix -F is used to indicate that its aim is to manage relatively fast changes. In CAM-V, a time-dependent baseline is created (unit BL), as well as a smoothed version of the cough count *B* using a temporal smoother (TS). The smoothing reduces the noise. The smoothed signal is compared to the baseline signal in a decision unit (DU). If the smoothed signal is too far beyond the baseline twice in three consecutive days, an alert is raised.

The proposed additional alert mechanism, CAM-F, involves checking for a consistent increase (in unit CI) and the total short-time increase (in unit TI). The outputs of both units are combined by a logical -AND operation (∧). In other words, if the cough is monotonically increasing over a number of days *D*, with a total increase of at least X(D), then an alarm is raised. We set the number of days *D* to 3 or 4 because of the need for a fast-responding alert and in view of the fact that 2 consecutive increases are not considered to indicate consistency but rather a relatively frequent occurrence in case of uncorrelated events. Assuming that *D* consecutive increases in a series of uncorrelated drawings from the same distribution would have odds of ${\left(1/2\right)}^{D}$, restricting it to a total increase beyond its natural day-to-day variation further reduces the odds of a false alert.

### 2.4. Exacerbation and Alert Events

The data collection and primary analysis were conducted in a double-blind fashion to prevent investigator bias. The identification of exacerbation periods was performed without knowledge of the data (raw or analysed) from the cough monitor [20]. The cough count trends and alerts were created without knowledge of any patient data. Only after all data were collected and the exacerbation periods and alerts defined were data shared, and associations between alerts and exacerbation periods were ascertained as explained below [21].

The identification of exacerbation periods was patient-initiated. The start date of a moderate AE-COPD event was defined as the date that the participant reported starting steroids and/or antibiotics for their chest or the date that a prescription for steroids and/or antibiotics was issued, excluding the renewal of ‘just-in-case’ medications. The end date was when the participant reported taking their last dose of steroids and/or antibiotics. For severe AE-COPD, the start and end dates are defined by the start and end of treatment (as per moderate AE-COPD) or the duration of hospitalisation (whichever is longer).

An exacerbation is an event lasting several days and not treated as a series of (independent) exacerbation days. Alerts occur on consecutive days, and such an alert train was defined as the alert event. This was chosen as operating on a day-to-day basis requires the creation of a ground truth for each day, which is rather ambiguous for prodrome and convalescence and was therefore not pursued. There is also no obvious requirement regarding how long an alert train should last or that its duration should somehow be coupled to the length of an exacerbation. Instead, a single alert for an (upcoming) AE-COPD event is sufficient; the alert train’s onset is therefore the basic phenomenon.

**Figure 3:**
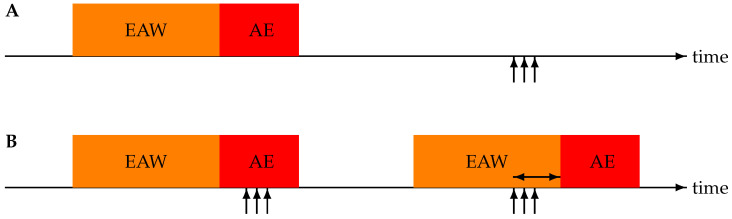
Illustration of the four possible associations between acute exacerbation (AE) periods and alert trains (represented by vertical arrows). Each AE is preceded by a 14-day early alert window (EAW). (**A**) Timeline with a missing alert (no start of an alert train during AE or EAW) and a false alert (alert train starting outside of AE and EAW). (**B**) Timeline with two detected exacerbations—the first one a late alert and the second one an early alert. The horizontal double arrow indicates the lead time.

An alert train is coupled to an exacerbation period if the alert sequence starts within the exacerbation period or before it, with a maximum of a 14-day difference. The former is called a late alert and the latter an early alert. The number of couplings defines the number of correct alerts. Alert trains and exacerbation periods without coupling are considered false and missing alerts, respectively. The time difference (days) between an early alert and exacerbation onset is called the lead time. Exacerbations starting in the first 13 days of monitoring were not taken into account—not only because of the association rule but also because CAM-V requires 13 days of initialisation for baseline creation [23]. For a graphical representation of the possible timings of exacerbation periods and alert trains, see Figure 3.

### 2.5. Evaluation Metrics

The first evaluation metric for the proposed system is the number of events, where an alert corresponds to an identified exacerbation event (i.e., sensitivity). The second one is the positive predictive value (PPV): the fraction of alerts that are considered true alerts. Since the alert mechanism is an event-based, one-class classifier (i.e., an (upcoming) non-exacerbation event does not exist [21]), the specificity, accuracy, and receiver operating characteristic are not defined. Instead, the frequency of false alerts is the pertinent metric: the number of alerts raised without an associated exacerbation relative to the total number of monitoring days. Additionally, the lead time is reported.

To strengthen the insight into the merits of the proposed system, we removed patients for which no increased coughing occurred during exacerbation periods. This was achieved in two ways: manual and automated selection. The automated approach was based on a logistic regression model with two readily available input parameters: age and the score on the COPD assessment test (CAT). The accuracy of this method was estimated at 85%; see [21].

To demonstrate the variability in patterns in the cough count, trend graphs *B* are shown, including raw data and smoothed curves, as generated by the unit TS inside CAM-V.

## 3. Results

**Figure 4:**
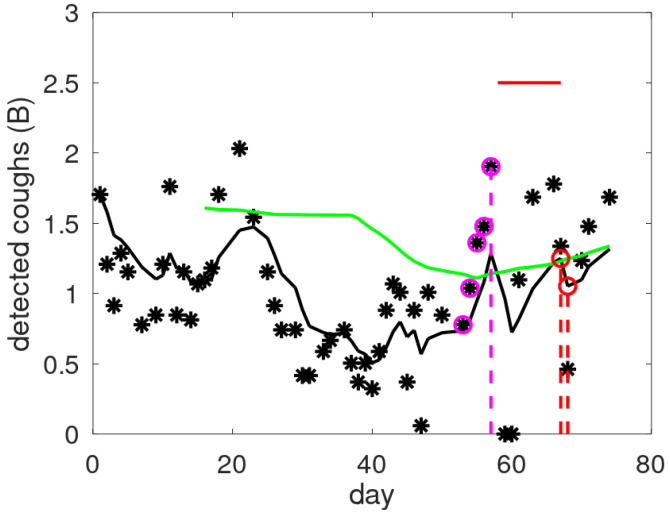
Examples of raw cough counts (asterisks), smoothed cough counts (black line), and threshold levels (green line) as a function of the monitored day. Alert days are indicated by a coloured dashed line for the validated mechanism (red) and the new one (magenta). The magenta circles provide the data giving rise to the alert in CAM-F: four consecutive increases with a total increase exceeding the threshold of 1 B. The red horizontal line indicates the exacerbation period.

Figure 4 contains an example showing cough counts with indications of exacerbation periods and alerts raised by CAM-V and CAM-F. A consistent increase in the raw cough count is clearly visible preceding the exacerbation. In this case, the new alert mechanism converts a late alert into an early alert, increasing the precision with which one can intervene at the onset of an exacerbation. The personalisation of individual alerts requires interpretation. For example, this patient had a severe exacerbation—note the two days of zero cough counts at the start of the exacerbation period, which correspond to a stay in the hospital.

The overall performance metrics comparing the baseline-oriented mechanism to the augmented alert mechanism are given in Table 1. The first row gives the results for CAM-V and covers the total cohort of 32 patients, with the data already reported in [20]. The two reduced patient sets both include 22 patients, but these cohorts are not the same. The automated selection process included two patients in whom the manual approach considered to have exhibited exacerbations without increased coughing. Obviously, the reduced cohort led to fewer observed exacerbations (manual selection (Ma.): 17; automated selection (Cl.): 21) and fewer monitored days. The percentage of missed exacerbations was clearly reduced with these selections, decreasing from 41% (11 out of 27) to 18% (Ma.: 3 out of 17) and 24% (Cl.: 5 out of 21).

Running the proposed alert scheme V + F creates two additional detections and a shift in the division between late and early detections (exemplified in Figure 4). Manual patient selection flags all but one exacerbation, while the automated patient selection procedure produces sensitivity of 86% (18 out of 21). Combining the outputs of CAM-V and CAM-F with a logical -OR operation increases the number of false alarms. For the false alarms, we use a range, as some alerts were considered as potential underdiagnosed exacerbations [20]. The number of false alarms for the reduced patient sets was almost doubled, leading to a rate of once per 6 to 10 months.

The 10 observed lead times in V + F mode are 0, 1, 6, 7, 7, 8, 8, 8, 9, and 12 days, meaning increased precision in detection: 80% of the early alerts have a lead time of more than 6 days.

**Figure 5:**
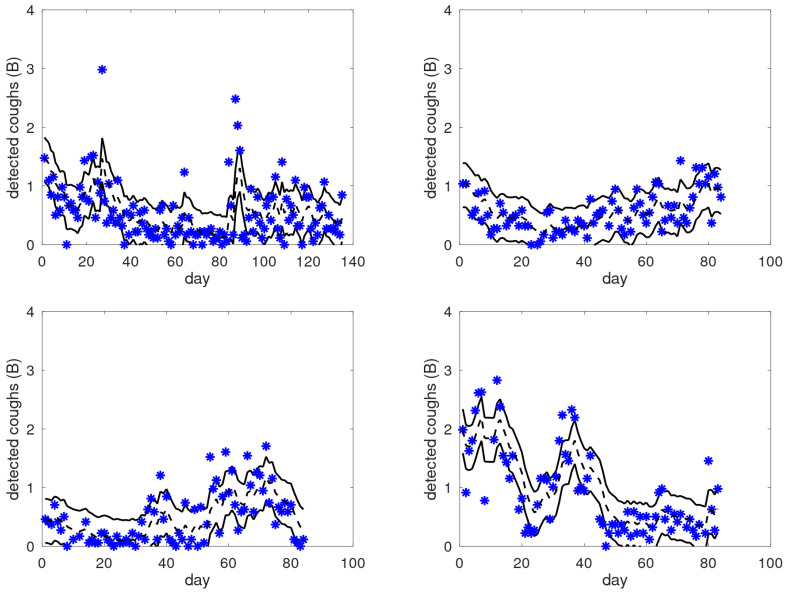
Examples of raw cough counts (asterisks), smoothed cough counts (black dashed lines), and dominant bands (between solid black lines) as a function of the monitored day for four patients from the target group. These graphs illustrate the large variability in the observed patterns across patients, moving from gradual and smooth transitions to sudden jumps.

The alert system is not merely a black box: traces of cough counts on the B-scale are interpretable. This implies that the patient may learn to recognise personal patterns in their cough counts and relate these to changes in behaviour and/or respiratory health. It also means that the alert functionality may benefit from these interpretations. To demonstrate these ideas, we consider Figure 5, where we plot cough count graphs plus a smoothed version (output of TS from CAM-V). Moreover, the filtered version is transformed into a band by adding +0.35 and −0.40 B; these offsets are first-order patient-independent estimates for the quartiles of the cough distribution [21]. For simplicity and clarity, the alerts and exacerbation periods are omitted. Note that the *y*-axis in the plots is uniform, ranging from 0 to 4 B.

Across these examples, we can see that the area bounded by the solid lines is indeed the area where most of the raw data points are located, independently of the level. We also observe significantly different behaviour in these patients; for instance, the top-right graph shows a consistent gradual increase from day 20 onward, whereas the bottom-right graph shows a fluctuating pattern. In the top-left graph, we can see clear and sudden outliers. Before and after the outliers around day 85, there is a clear change in the average level of coughing. In the bottom-left graph, we can see an elevated cough level after day 55, with frequent outliers. These observations raise the question of whether patients can identify a reflection of their respiratory status in these data and are able to interpret them. If so, this would open up new possibilities for personalised medicine: it may help patients to gain insights into (changes in) their situation, and cough count graphs that include patient annotations may offer a new source of information for the clinician, being characterised by a combination of objective and patient-reported data. In the long run, the annotation of events may be of benefit for the alert mechanism, e.g., it might be used for general improvements to the alert system (improved alert rules), or it may be used to personalise the mechanism to the specific patient.

**Figure 6:**
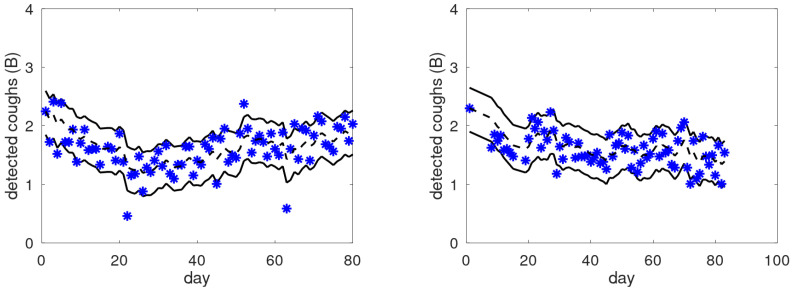
Examples of raw cough counts (asterisks), smoothed cough counts (black dashed lines), and dominant bands (between solid black lines) as a function of the monitored day for two COPD patients that are not in the target group.

In contrast, Figure 6 shows two graphs of the cough trends of COPD patients not belonging to the target population. Although the cough trends show long-term trends, there are no obvious cues as to well-defined periods with increased coughing that could correspond to an exacerbation, despite the fact that both patients experienced an exacerbation during the monitoring period. This suggests that these graphs display no phenomena from which the patient could extract insights or improve their self-management.

## 4. Discussion

The validated alert mechanism, CAM-V, was designed based on the notion that day-to-day cough variation is high and that temporal smoothing is a means to mitigate this, thereby obtaining a better input signal for trend and baseline analysis. Smoothing has drawbacks in the sense that it is unable to follow fast changes. It thus exhibits less sensitivity to such events and a delayed response. The proposed additional alert mechanism, CAM-F, complements the validated one to improve the precision, where the large day-to-day variation is addressed via the requirement for a consistent and large increase.

Although the day-to-day variability in cough counts is large [25,26,27], our study shows that, with careful data processing, relevant clinical information can be extracted from it. The alert mechanism is a simple rule-based procedure, yet most exacerbations were captured, including early alerts with large lead times. Uniform processing with patient-independent settings in the alert proved feasible owing to the mapping of counts to the B-scale. No use has been made of machine learning in the alert mechanism; only simple intuitive rules have been used. The risk of overfitting and the generalisation issues often encountered when applying machine learning to small datasets have been averted.

The addition of a new alert component via a logical -OR operation increases the number of false alerts. In the case of automated patient selection, there were 11 alerts not associated with an exacerbation—one was at the very end of the trial period, and, therefore, the associated exacerbation may have occurred later. From the remaining 10, five were potentially relevant respiratory events, as the cough count remained high for a number of consecutive days. We therefore argue that approximately 5–10 truly false alerts were raised, meaning a false alarm rate of between one and two per year for the reduced cohort. For practical operation, this is considered a manageable burden.

Regarding the positive predictive value for classifier-based patient selection and the full system (V + F), it was within the range of 65–78% (18 out of 28 to 18 out of 23; see Table 1). In other words, the majority (2/3 or more) of alerts are meaningful and considered to contribute to acceptance and adherence.

For clarity, we also mention that the definition of exacerbation onset—and, therefore, the subdivision of late/early detection and lead times—was complicated by the fact that the identified exacerbations were patient-initiated. This may lead to underdiagnosis [28] and has partly been addressed by using a false alarm range instead of a single value. In addition to this, the exacerbation ground truth may be biased, as patients were asked to fill out a daily questionnaire. The effect of such an activity on patient-initiated exacerbation identification is unclear. For completeness, we provide a causal rule-based mechanism for the questionnaire data in Appendix A. The results indicate that it is unlikely that these questionnaire data enable high-quality performance in AE-COPD prediction.

The proposed cough-based alert mechanism was designed based on available data. The number of exacerbations was low, prohibiting the fine-tuning of the parameters. Validation and parameter fine-tuning require new experiments, and, with sufficient data, artificial intelligence (AI) approaches to merge the designed rule-based methods may be advantageous to obtain a more flexible model.

## 5. Conclusions

In a post hoc analysis, the cough trend data of a stratified cohort of COPD patients were considered. A proposal for an addition to a previously validated alert mechanism for COPD exacerbations was created and tested based on existing data. This combination provides personalised data and increased precision, demonstrated by increased sensitivity and an increased fraction of early alerts. For early alerts, in the target population, there is mostly a considerable lead time (6 days or more). The false alarm rate seems manageable for practical operation (about 1.5 false alerts per year). In addition to this, the system is a rule-based classifier with interpretable signals. Patients in the target group may benefit from the cough trend visualisation, obtaining personalised insights into their respiratory health and adverse conditions. The annotation of the trend graphs by a patient may be useful for diagnostic purposes. For a telehealth application deploying the alert mechanism, stratification is essential for a range of (related) reasons, such as achieving increased precision in performance, creating trust in the system, maintaining adherence, generating personalised insights into cough patterns, and improving self-management. The proposed system needs validation in independent cohorts to validate the preliminary performance results obtained in this post hoc analysis. Further study of the alert mechanism could target various hypotheses: the benefit of better management for the patient, improved exacerbation recovery through early alerts, or reduced hospitalisation.

The novelty of this study lies in two aspects. Firstly, it demonstrates that the performance of the validated cough-based AE-COPD mechanism can be improved by various strategies—in particular, stratification and novel processing—thereby boosting the precision. Secondly, cough count graphs reveal details that have not yet been studied and suggest that patient annotation may provide new insights that are beneficial for self-management and diagnosis.

## Figures and Tables

**Table 1:** Performance for original and stratified cohorts. Sel.: patient selection, where Ma. indicates the manual and Cl. the classifier-based selection, both originating from [21]. The ’CAM’ column indicates the alarm mechanism, where the validated mechanism CAM-V is used either alone or in conjunction with the additional component CAM-F (indicated as V + F). The acute exacerbations (’AE’) column gives the total number of exacerbations. The correct detections are subdivided into early and late alerts, as illustrated in Figure 3. The ’False’ column indicates the number of false alerts (i.e., without associated exacerbations), which is given as a range, as some exacerbations may have gone unnoticed (see [20]).

Settings					Alerts				
**Sel.**	**CAM**	**Patients**	**Days**	**AE**	**Missing**	**Correct**	**Early**	**Late**	**False**
–	V	32	2694	27	11	16	7	9	4–7
Ma.	V	22	1944	17	3	14	7	7	3–6
Cl.	V	22	1817	21	5	16	7	9	3–6
–	V + F	32	2694	27	9	18	10	8	6–11
Ma.	V + F	22	1944	17	1	16	10	6	5–10
Cl.	V + F	22	1817	21	3	18	10	8	5–10

## Data Availability

The data presented in the study are stored securely at Hull York Medical School. The investigators act as custodians for the data processed and generated in the study, and they are also responsible for access to any information included. Requests can be made to A.H. Morice. Due to privacy and institutional regulations, the data are not publicly accessible.

## Abbreviations

The following abbreviations are used in this manuscript:

AE	Acute Exacerbation
CAM	Cough-Based Alert Mechanism
CAM-F	Fast-Response CAM
CAM-V	Validated CAM
CAT	COPD Assessment Test
COPD	Chronic Obstructive Pulmonary Disease

## Appendix A. AE-COPD Alert Performance for Questionnaire Data

The daily questionnaire used in the trial was taken from [29]. It was found that the original alert rules were not suitable considering how the patients had completed the questionnaires: among the 3773 records, there were 1244 alerts generated [20]. In the same paper, an adaptation of the rules was considered, where the detection rules were applied to the questionnaire score after the subtraction of the median. This improved the results considerably. However, as a result, a non-causal operation was introduced. Here, we consider a variant where a causal mechanism is used to perform a conceptually similar operation. The subtraction of the median is replaced by the subtraction of a short-term median, which is created in the same way as the baseline cough count in the validated mechanism, CAM-V, i.e., the short-term median is taken over the first 11 days of the most recent fortnight. As with the cough count mechanism, this means that there is an initialisation period of 13 days, and the alert mechanism does not operate on these days. Alerts are grouped into alert trains, and the identification of alert trains and exacerbation periods was performed. Note that counting false alert days instead of false alert trains would provide a considerably larger false alarm rate.

**Table A1:** Performance of two alert mechanisms on the questionnaire data: the non-causal from [20] and its adaptation to a causal one (see text). AE stands for acute exacerbation, being divided into detected (correct alert) and missed ones. Correct alerts are further divided into early and late alerts. The ’False’ column indicates the number of false alerts.

				Alerts				
**System**	**Patients**	**Days**	**AE**	**Missing**	**Correct**	**Early**	**Late**	**False**
Non-causal	38	3773	40	12	28	18	10	42
Causal	38	3279	35	16	19	16	3	47

The results for both the causal and non-causal mechanisms are given in Table A1. The causal mechanism provides poorer performance. From an overall perspective, the sensitivity is 54–70% (19 out of 35–28 out of 40), the false alarm rate is 4.5–5.2 per year, and the positive predictive value is 29–40% (28 out of 70–19 out of 66). The low PPV and high false alarm rate for both constructed mechanisms suggest that intuitive, simple rules show limited success in retrieving the desired AE-COPD information, if present at all.
